# Volume of interest-based [^18^F]fluorodeoxyglucose PET discriminates MCI converting to Alzheimer's disease from healthy controls. A European Alzheimer's Disease Consortium (EADC) study

**DOI:** 10.1016/j.nicl.2014.11.007

**Published:** 2014-11-18

**Authors:** M. Pagani, F. De Carli, S. Morbelli, J. Öberg, A. Chincarini, G.B. Frisoni, S. Galluzzi, R. Perneczky, A. Drzezga, B.N.M. van Berckel, R. Ossenkoppele, M. Didic, E. Guedj, A. Brugnolo, A. Picco, D. Arnaldi, M. Ferrara, A. Buschiazzo, G. Sambuceti, F. Nobili

**Affiliations:** a Institute of Cognitive Sciences and Technologies, Consiglio Nazionale delle Ricerche (CNR), Rome, Italy; b Department of Nuclear Medicine, Karolinska Hospital, Stockholm, Sweden; c Institute of Bioimaging and Molecular Physiology, Consiglio Nazionale delle Ricerche (CNR), Genoa, Italy; d Nuclear Medicine, Department of Health Sciences (DISSAL), University of Genoa, IRCCS AOU San Martino-IST, Genoa, Italy; e Department of Hospital Physics, Karolinska Hospital, Stockholm, Sweden; f National Institute for Nuclear Physics (INFN), Genoa, Italy; g LENITEM Laboratory of Epidemiology and Neuroimaging, IRCCS S. Giovanni di Dio-FBF, Brescia, Italy; h University Hospitals and University of Geneva, Geneva, Switzerland; i Neuroepidemiology and Ageing Research Unit, School of Public Health, Faculty of Medicine, The Imperial College London of Science, Technology and Medicine, London, UK; j West London Cognitive Disorders Treatment and Research Unit, London, UK; k Department of Psychiatry and Psychotherapy, Technische Universität, Munich, Germany; l Department of Nuclear Medicine, Technische Universität, Munich, Germany; m Department of Nuclear Medicine & PET Research, VU University Medical Center, Amsterdam, The Netherlands; n APHM, CHU Timone, Service de Neurologie et Neuropsychologie, Aix-Marseille University, INSERM U 1106, Marseille, France; o APHM, CHU Timone, Service de Médecine Nucléaire, CERIMED, INT CNRS UMR7289 , Aix-Marseille University, Marseille 13005, France; p Clinical Neurology, Department of Neuroscience, Rehabilitation, Ophthalmology, Genetics, and Mother-Child health (DINOGMI), University of Genoa, IRCCS AOU, San Martino-IST, Genoa, Italy

**Keywords:** MCI, FDG-PET, Volume of interest, Discriminant analysis, EADC

## Abstract

An emerging issue in neuroimaging is to assess the diagnostic reliability of PET and its application in clinical practice. We aimed at assessing the accuracy of brain FDG-PET in discriminating patients with MCI due to Alzheimer's disease and healthy controls. Sixty-two patients with amnestic MCI and 109 healthy subjects recruited in five centers of the European AD Consortium were enrolled. Group analysis was performed by SPM8 to confirm metabolic differences. Discriminant analyses were then carried out using the mean FDG uptake values normalized to the cerebellum computed in 45 anatomical volumes of interest (VOIs) in each hemisphere (90 VOIs) as defined in the Automated Anatomical Labeling (AAL) Atlas and on 12 meta-VOIs, bilaterally, obtained merging VOIs with similar anatomo-functional characteristics. Further, asymmetry indexes were calculated for both datasets. Accuracy of discrimination by a Support Vector Machine (SVM) and the AAL VOIs was tested against a validated method (PALZ). At the voxel level SMP8 showed a relative hypometabolism in the bilateral precuneus, and posterior cingulate, temporo-parietal and frontal cortices. Discriminant analysis classified subjects with an accuracy ranging between .91 and .83 as a function of data organization. The best values were obtained from a subset of 6 meta-VOIs plus 6 asymmetry values reaching an area under the ROC curve of .947, significantly larger than the one obtained by the PALZ score. High accuracy in discriminating MCI converters from healthy controls was reached by a non-linear classifier based on SVM applied on predefined anatomo-functional regions and inter-hemispheric asymmetries. Data pre-processing was automated and simplified by an in-house created Matlab-based script encouraging its routine clinical use. Further validation toward nonconverter MCI patients with adequately long follow-up is needed.

## Highlights

• ^18^F-FDG-PET/CT analysis of metabolic differences between MCI converting to AD and HC
• Large and very well controlled cohorts from EADC-Consortium were investigated.
• Data were analyzed by a friendly-to-use Matlab-based script and Support Vector Machine.
• Excellent discrimination between MCI and HC (sensitivity 92%; specificity 91%)
• Highest accuracy reported so far in MCI and promising implementation in clinical routine

## 1 Introduction

[^18^F]Fluorodeoxyglucose PET (FDG-PET) is one of the neurodegeneration biomarkers included in the new research criteria for the diagnosis of Alzheimer's Disease (AD) by the International Working Group (IWG) in 2007 and 2010 (Dubois et al., 2007; Dubois et al., 2010) and in the new diagnostic criteria of AD by the National Institute of Aging–Alzheimer Association (NIA–AA) (McKhann et al., 2011). Notably, FDG-PET induced substantial changes in the diagnosis and pharmacological management of patients with dementia, and in recognizing AD among atypical cases (Laforce et al., 2010). Moreover, FDG-PET has been included in the NIA–AA diagnostic criteria of Mild Cognitive Impairment (MCI) due to AD (Albert et al., 2011; Sperling et al., 2011) while the recently proposed IWG-2 research criteria hypothesize its role as a disease evolution rather than as a pure diagnostic biomarker (Dubois et al., 2014). All these new criteria are based on evidence accumulated since 1984 (McKhann et al., 1984) but need now to be applied and verified, i.e., validated, in large patient populations. This process is ongoing and available data are indeed encouraging (Lucignani and Nobili, 2010).

However, an emerging issue, as well as for atrophy indexes with Magnetic Resonance Imaging (MRI), is how to measure or evaluate the information contained in the FDG-PET scans' data to be used in clinical routine at the individual level. The metrics chosen to evaluate hypometabolism may carry variability in accuracy as high as the difference in accuracy between different biomarkers (Frisoni et al., 2013). The commonest way is the visual reading that is the cornerstone of any report but it may not be accurate enough (Foster et al., 2007; Patterson et al., 2010) particularly at the early stages of the disease (i.e. MCI) or when expert readers are not available on site. For this reason, some automated software, either free on the web (such as Statistical Parametric Mapping, SPM, and 3D-Stereotactic Surface Projections, 3D-SSP) or traded on the market (such as the T-sum computation and the PALZ score embedded in PMOD®) been applied to analyze patients' scans. Such as for T-sum, accuracy may vary between patients with AD-dementia and patients with MCI, as it has been shown for MRI (Chincarini et al., 2014). Machine learning and pattern recognition algorithms have also been developed to aid in neuroimage analyses — for a review see Lemm et al. (2011).

Recently, using various automated image-based classification methods, efforts have been made to discriminate AD and MCI patients from healthy controls by MRI (Cuingnet et al., 2011), FDG-PET (Arbizu et al., 2013; Gray et al., 2012; Illan et al., 2011; Toussaint et al., 2012), in a multimodal fashion (Zhang et al., 2011) or implementing univariate and multivariate analyses (Toussaint et al., 2012), cross-sectional and longitudinal information (Gray et al., 2012), image projection onto a feature space (Illan et al., 2011) and atlas- (Cuingnet et al., 2011) and manually- (Zhang et al., 2011) based segmentation methods.

Most of these studies were conducted in large cohorts of patients from the Alzheimer's Disease Neuromaging Initiative (ADNI) database and reached a statistical accuracy in discriminating AD patients from controls of 93% when using three biomarkers (MRI, FDG-PET and CSF) (Zhang et al., 2011) and higher than 90% with FDG-PET only (Arbizu et al., 2013; Gray et al., 2012; Illan et al., 2011). However accuracies were substantially lower in studies in which MCI (later converted to AD) was considered instead of patients with full-blown AD dementia (Cuingnet et al., 2011; Toussaint et al., 2012; Zhang et al., 2011). Though FDG-PET has been shown to correlate with the severity of cognitive impairment (Chen et al., 2010), subtle abnormalities at the earliest (prodromal) stages of the disease may be more difficult to be detected. Yet, it is exactly at these stages that precise diagnosis and, possibly, prognosis are needed.

In fact, although effective disease-modifying drugs are still in the pipeline, there is no doubt that these should be used as earlier as possible in the natural history of the disease since a critical issue in MCI is the possible conversion to AD and biomarkers able to predict this event with high accuracy would allow for all possible therapeutic intervention to be immediately implemented. As a consequence, the need of an early diagnosis in faint symptomatic patients is mandatory and even in the era of amyloid-PET, the demonstration of synaptic dysfunction at specific brain sites maintains a high positive predictive value (Dukart et al., 2013). In the case of AD the validation of methodologies aiming at predicting disease progression should be performed in two different stages: (i) assessment of the capability to differentiate normal aging from MCI; and (ii) assessment of the capability to predict the conversion of MCI to AD.

In the present study we aimed at evaluating the accuracy of a method implementing automated Volume of interest (VOI)-based segmentation and Support Vector Machine in discriminating a large sample of patients with MCI who later converted to AD from healthy controls. All clinical-neuropsychological diagnosis and FDG-PET have been collected in five centers of the European Alzheimer's Disease Consortium (EADC). The accuracy of the present method was evaluated by comparison with an already validated discriminating technique (Herholz et al., 2002) by the application of PALZ scoring to the same sample of patients and controls.

## 2 Materials and methods

### 2.1 Subjects

Patient and control selection and definition as well as PET technical details in the five EADC centers have been reported in previous papers (Albert et al., 2011; Morbelli et al., 2012; Morbelli et al., 2013). Local ethics committee approved the project and all subjects signed the informed consent. Briefly, patients with amnestic MCI according to the current criteria (Albert et al., 2011) were followed by clinical-neuropsychological assessment and only those developing dementia of the AD type during the observation period (mean conversion time 22.6 ± 16.0 months, range 6–42) were considered for this study (MCI-converters). These were sixty-two patients, 34 women and 28 men, age range: 54–86 years (mean: 72.3 ± 8.2); mean MMSE score at the time of FDG-PET 27.0 ± 1.5. One hundred and nine healthy subjects investigated in the same centers, including 57 women and 52 men (age range: 52–83 years, mean: 66.8 ± 6.5; mean MMSE score at the time of FDG-PET: 29.3 ± 1.0) served as controls. Their health status was checked again with a clinical interview about 1 year later (mean: 11.8 ± 4.8 months). Having relatives affected by dementia was not an exclusion criterion for healthy controls. For both patients' and controls' groups, MRI evidence of major stroke or brain mass was considered as an exclusion criterion, while white matter hyperintensities, leucoaraiosis and lacunae did not constitute an exclusion criterion if the Wahlund score was <3 in all regions (Wahlund et al., 2001). Drugs known to interfere with brain metabolism and perfusion were slowly tapered and withdrawn whenever possible, before undergoing neuropsychological and FDG-PET examinations. This was successfully achieved in most patients and controls, with just 14 patients and 8 controls taking stable doses of selective serotonin reuptake inhibitors and none taking benzodiazepines. Mean age was significantly different between patients and controls. In order to take physiological effects of age and gender on PET data into consideration, and to distinguish these effects from changes due to pathological condition, the variations of PET data as function of age and gender were analyzed in controls.

### 2.2 Image acquisition and preliminary analysis

FDG-PET was performed in all centers, according to the European Association of Nuclear Medicine guidelines (Varrone et al., 2009). Injection, acquisition and image reconstruction protocols are detailed in a previous paper of the EADC study group (Morbelli et al., 2012). DICOM files were exported and converted into Analyze format. At a preliminary step, MCI-converter patients were compared with controls by Statistical Parametric Mapping (SPM8) (Friston et al., 1994) in order to verify the well known pattern of posterior cingulated and temporoparietal hypometabolisms in early AD. To avoid inconsistencies deriving from the use of the default SPM brain H_2_O template (Gispert et al., 2003), PET scans were normalized using a customized brain FDG PET template, obtained from brain PET and MR imaging scans of 27 healthy subjects as detailed elsewhere (Morbelli et al., 2012). After template editing, all brain PET scans were processed by affine and nonlinear spatial normalization into the stereotaxic space of the Montreal Neurological Institute through the study-customized FDG template using SPM. The spatially normalized sets of images were then smoothed with an 8-mm isotropic Gaussian filter to blur individual variations in gyral anatomy and to increase the signal-to-noise ratio. Brain PET from MCI-converter patients were compared on a voxel-by-voxel basis to those from the normal controls using a “two-sample t-test” design of SPM8 (Friston et al., 1994) implemented in Matlab R2014a (MathWorks, Natick, Massachusetts, USA). The significance threshold was set at p < 0.05, corrected for multiple comparisons with family wise error (FWE). Only clusters containing more than 100 voxels were considered to be significant. Age, sex and center were included in the analysis as confounding variables.

### 2.3 Region of interest identification and data preprocessing

Mean FDG uptake values were computed in 45 anatomical volumes of interest (VOIs) in each hemisphere (90 VOIs) as defined by the AAL Atlas (Tzourio-Mazoyer et al., 2002).

The dataset to be analyzed was obtained by an in-house created Matlab-based script that automatically processed mean FDG uptake signal intensity from each of the 90 AAL VOIs. The mean signal intensities computed for each VOI were normalized within each subject to the average intensity of the cerebellar VOIs included in AAL as defined by Schmahmann et al. (1999). This choice was based on the knowledge that the cerebellum is poorly affected by the AD pathological process and on the evidence that, when using the cerebellum instead of whole brain counts as the reference region, accuracy in distinguishing AD patients from controls increases (Soonawala et al., 2002). Next, the number of VOIs was further reduced by merging regions with similar anatomo-functional characteristics into meta-VOIs in order to decrease the number of variables for statistical analysis and to verify if the analysis of specific and functionally meaningful meta-VOIs might contribute to characterize the pathological process. All these calculations were performed by the Matlab script in a single step allowing a substantial spare of time and simplifying the whole process. Twelve meta-VOIs were constructed in each hemisphere: 1. Occipital Cortex (Calcarine/Lingual/Inferior Occipital/Middle Occipital/Superior Occipital Gyri); 2. Thalamus/Putamen/Pallidum/Caudate; 3. Parahippocampal gyrus/Amygdala/Hippocampus/Insula; 4. Orbito-frontal Cortex (Inferior Frontal/Medial Frontal/Superior-Orbital Frontal Gyri); 5. Frontal Cortex (Middle Frontal/Superior Frontal/Superior-Medial Frontal Gyri); 6. Cuneus/Fusiform Gyrus/Precuneus; 7. Postcentral Gyrus/Precentral Gyrus/Supplementary Motor Area; 8. Parietal Lobe (Inferior Parietal/Superior Parietal Gyri); 9. Anterior Cingulate Gyrus, 10. Posterior Cingulate Gyrus, 11. Temporal Lobe (Inferior Temporal/Middle Temporal/Superior Temporal Gyri), and 12. Temporal Pole (Middle Temporal Pole/Superior Temporal Pole Gyri).

Data from control subjects were first analyzed by applying a General Linear Model (GLM) to evaluate the effect of age and gender and consequently correct FDG-PET analyses accordingly. This analysis was restricted to controls in order to separate possible physiological weakening of metabolic activity with age (and gender differences) from the effect of pathological conditions whose probability increases with age. Discriminant analysis was performed by a non-linear classifier based on the Support Vector Machine (SVM) method with Radial Basis Functions (Cortes and Vapnik, 1995). The procedure was independently applied to the different datasets extracted from the same groups of PET images in order to evaluate the influence of data preprocessing and to identify the regions with the highest statistical impact. Each dataset included cerebellum-normalized FDG-PET values relevant to a particular set of VOIs. Since asymmetric metabolism has often been found in pathological condition (Kovalev et al., 2006; Rodriguez et al., 1993; Zahn et al., 2004), absolute values of inter-hemispheric asymmetries were also considered and evaluated, for each VOI, as:

$$asy_{voi}=\frac{\mathrm{abs}\left(nv_{voi,l}-nv_{voi,r}\right)}{nv_{voi,l}+nv_{voi,r}}$$

where *nv*_voi,x_ is the normalized FDG value relevant to the right (r) or left (l) hemisphere and abs() stands for the absolute value.

The two basic datasets were made up by the normalized values relevant to the 90 (45 bilateral) AAL regions and by the normalized values relevant to the 24 (12 bilateral) anatomo-functional homogeneous meta-VOIs. Two other datasets were obtained by adding hemispheric values to each of the previous ones: 90 VOIs plus 45 asymmetry values for the AAL regions (135 values) and 24 VOIs plus 12 asymmetry values for the meta-VOIs. This last dataset (36 values) was then the starting point to explore the accuracy of a smaller set of variables by the application of a step-wise backward selection procedure (by removing the less influential variables, one at a time).

In order to evaluate our VOI-based classifier with reference to an already validated system, the PMOD software (PMOD Technologies, http://www.pmod.com) was used for automatic, voxel-based evaluation of scans with the ‘Alzheimer’ option computing the ‘Probability of ALZheimer’ or PALZ score. The PALZ score (Herholz et al., 2002) is a voxel-based parametric mapping method yielding diagnostic information on brain regions that are typically affected in AD: it aims at discriminating AD from healthy controls above 50 years of age. Individual FDG-PET images are compared to a fixed database of normal elderly scans through a voxel-wise t-test, including age as confounding variable. The PALZ score is computed as the sum of t-scores in a pre-defined AD-pattern mask and is compared with a threshold for abnormality drawn from the normal elderly database: PALZ-score higher than threshold (11,089) indicating abnormal FDG-PET. As some centers provided DICOM files and some others provided Analyze files, DICOM files were converted into Analyze format. In fact, the use of different formats gives rise to slight differences in PALZ scores, as already outlined in a previous paper (Caroli et al., 2012). Preliminary to the PALZ score computation, scans in Analyze format were checked for display orientation and eventually reoriented to correct anatomic position.

### 2.4 Statistics

The reliability of the discrimination was evaluated by a series of conventional parameters. For each classification procedure (PALZ-score and VOI-based with different datasets) we evaluated accuracy, sensitivity, specificity, positive and negative likelihood ratios, odds ratio and the area under the Receiver Operating Characteristic (ROC) curve (AUC). All these parameters were evaluated by a cross-validation procedure which entails the separation between the model fitting and testing to enable generalization of results and to prevent overfitting (the adaptation to features peculiar only to the study sample). Cross-validation was implemented by the ‘leave-one-out’ technique in which each subject was classified by a model fitted to all remaining ones, so creating a virtually-independent testing set with the same size of the original sample. As a by-product of the SVM training we could calculate the ranking for each region, that is a rough measure of how relevant is a particular feature to the outcome of the classifier.

All parameters measuring discrimination capability were estimated with their 95% confidence intervals (CIs) which were computed according to the characteristics of each parameter. The Wald interval with exact binomial probabilities was applied to sensitivity, specificity and accuracy (Brown et al., 2001); CIs for positive (+LR) and negative (−LR) likelihood ratios were estimated according to the Simel method (Simel et al., 1991); CIs for the odds ratio were estimated by the conventional log-transform method (Glas et al., 2003) while CIs for the ROC-AUC were estimated by the bootstrap method described by Qin and Hotilovac (2008). For a comparison between the results obtained with different procedures, the ROC-AUCs were compared by evaluating the z-value for paired scores according to Hanley and McNeil (1983).

Discriminant analysis by SVM and following ROC curve analysis and accuracy measurements were performed by using the Statistics Toolbox of Matlab R2014a (MathWorks, Natick, Massachusetts, USA).

## 3 Results

**Fig. 1:**
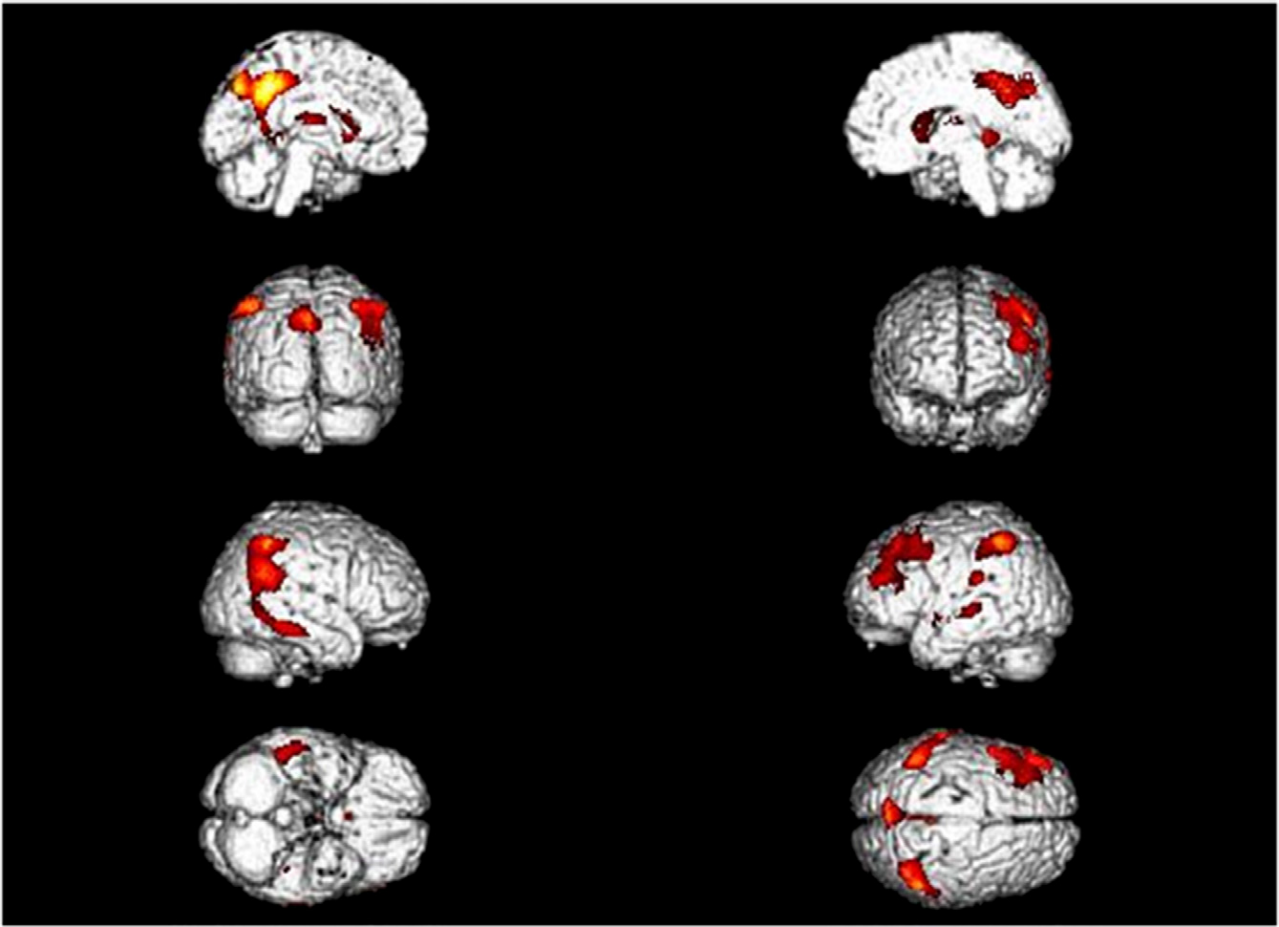
Metabolic differences between healthy controls and MCI patients converting to AD. Tree-dimensional rendering of SPM analysis showing those regions in which ^18^F-FDG uptake was significantly lower in MCI-converters(n = 62) than in healthy controls (n = 109) (threshold p < 0.05, corrected for multiple comparisons with the family-wise error (FWE) option). Top row left: medial left view; top row right: medial right view; second row left: posterior view; second row right: frontal view; third row left: right-side view; third row right: left-side view; bottom row left: view from below; and bottom row right: view from above. Talairach coordinates and further details are provided in Supplementary Table e1.

In the preliminary comparison of whole brain FDG values at the group level using SPM8, relative hypometabolism was found in the bilateral precuneus, and posterior cingulate, temporo-parietal and frontal cortices, as expected (Fig. 1, Supplementary Table 1).

GLM analysis of normalized regional FDG values in the control subjects as a function of age and gender showed a significant effect of region, and region × gender interaction for both the partition into 90 AAL regions (region effect: F_89,9434_ = 11.42, p < 0.0001; region × gender interaction: F_89,9434_ = 5.18, p < 0.0001) and for the partition into 24 meta-VOIs (region effect: F_23,2438_ = 11.34, p < 0.0001; region × gender interaction: F_23,2438_ = 6.67, p < 0.0001). No significant effect was found for age or for the age × region interaction. Normalized regional values were accordingly corrected for the effect of gender.

Discriminant analysis by SVM enabled the classification of subjects with an accuracy ranging between .91 and .83 as a function of data organization (Table 1). The best values were obtained from the 90 AAL regions (90R) and from a subset of 6 meta-VOIs plus 6 asymmetry values (6R + 6A). Relatively less accurate classification was obtained with the 24 meta-VOIs (24R) and with the 90 AAL regions with associated asymmetry values (90R + 45A). As for the subset 6R + 6A, which provided the overall best classification (sensitivity: .92, specificity: .91), the relevant AAL regions were ranked: 1. Parahippocampal Gyrus/Amygdala/Hippocampus/Insula (left), 2. Frontal Cortex (left), 3. Postcentral Gyrus/Precentral Gyrus/Supplementary Motor Area (left), 4. Parietal Cortex (right), 5. Anterior Cingulate (left), and 6. Posterior Cingulate (left). In the same subset, the most relevant AAL regions for asymmetry values were: 1. Occipital Cortex, 2. Pallidus/Caudate nucleus/Thalamus, 3. Parahippocampal Gyrus/Amygdala/Hippocampus/Insula, 4 Orbito-frontal Cortex, 5. Frontal Cortex, and 6. Cuneus/Fusiform/Precuneus.

The high rate of correct classification, particularly for the datasets 90R and 6R + 6A, was emphasized by the high positive likelihood ratios (the ratio between the probabilities of positive test in patients with respect to controls) and low negative likelihood ratios (the ratio between the probabilities of negative test in patients with respect to controls) and by the high diagnostic odds ratios (the ratio between the odds of positivity in patients with respect to controls). The values of these parameters are reported in Table 1 with relevant CIs showing a large overlapping among the different datasets.

The same measures (with relevant confidence intervals) are reported for the classification based on the PALZ-score. We first considered the classification based on the standard fixed threshold (PALZ-score = 11,089) which yielded a high specificity (.92) but a relatively low sensitivity for MCI-converter patients. A different threshold (8116) drawn from the point on the ROC curve closer to the optimal one (sensitivity = specificity = 1) yielded a higher sensitivity (.77) but lower specificity (details in Table 1).

**Fig. 2:**
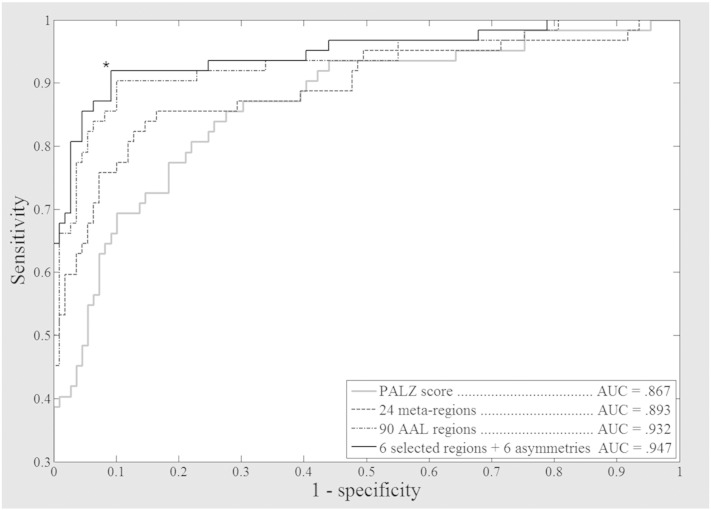
Receiver Operating Characteristic curves: classifier comparisons. Receiver operating characteristic (ROC) curves obtained by PALZ discrimination tool and by Support Vector Machine classifier as applied to 3 different datasets drawn from the same set of neuroimages: 90R: 90 AAL regions; 24R: 24 anatomo-functional meta-regions; and 6R + 6A: 6 meta-regions and 6 inter-hemispheric asymmetries drawn from step-wise backward selection. The best classification, marked by an asterisk, was obtained by a subset of 6 meta-regions and 6 asymmetry values, yielding 92% sensitivity and 91% specificity. Both 6R + 6A and 90R performed significantly better than PALZ-score (6R + 6A: p < 0.02; 90R: p < 0.05).

The ROC curves relevant to the PALZ-score and 3 of the analyzed datasets (90R, 24R and 6R + 6A) are showed in Fig. 2. Pairwise comparisons between the VOI-based procedures and PALZ-score showed that the procedure based on the best fitted meta-regions (6 selected regions + 6 asymmetries) performed significantly better than the PALZ-score (greater AUC: z = 2.14, p < 0.02) and the same was true for the initial 90 ALL regions (z = 1.68, p < 0.05) while the difference between the 24-meta-regions and PALZ classification was not significant (z = 0.61, N.S.).

## 4 Discussion

Our data confirm and strengthen not only the value of FDG PET analysis as a valuable tool in the diagnostic process for AD but also the capability of automated classifiers to accurately identify the disease at the stage of MCI, in our sample on average 2 years before conversion.

In a clinical setting in which neuroimaging is considered to be a supportive feature, effective tools for automatic identification of AD-related hypometabolic patterns at the individual level are needed. In this respect, the relevance of automated image-based classifications in clinical routine increases if the implemented method will overcome the accuracy of 90%, reached by the consensus diagnostic criteria for AD as validated against neuropathology (Ranginwala et al., 2008), which is especially of value if AD is still in its prodromal phase, i.e. MCI. In our study all 62 MCI patients converted to AD after a mean follow-up time of about 2 years allowing to compare our discrimination values to a robust gold standard, although if not to neuropathology. Furthermore, the recruited subjects were carefully stratified by different centers undergoing neuropsychological assessment. This accurate selection has decreased the possibility for the results to be biased by variables out of control, notably the lack of conversion and stable or improving memory function in non-converting MCI patients (Pagani et al., 2010). In addition, the metabolic differences found in the preliminary analysis between the two cohorts (Fig. 1) are fully consistent with the prevalent literature on the metabolic impairment in MCI (see Bohnen et al., 2012 and Nobili et al., 2014 for review) supporting the appropriateness of group selection and the reliability of the classification analyses.

The proposed method shows an accuracy of 91% in discriminating MCI-converters from healthy controls with an area under the ROC curve of almost 95% being superior to previous investigations in MCI patients applying similar techniques based on unimodal biomarkers and reporting sensitivity and specificity slightly higher than 80% (Salas-Gonzalez et al., 2010; Toussaint et al., 2012).

In this respect the accuracy of our analyses is comparable to those of investigations seeking to classify AD-dementia and healthy controls (Bloudek et al., 2011; Herholz et al., 2002; Ishii et al., 2006; Kakimoto et al., 2011; Lehman et al., 2012), even when multimodal biomarkers were combined (Zhang et al., 2011).

We compared our results with the ones obtained by the PALZ-score, a procedure which was applied to discriminate AD patients from healthy elders with high sensitivity and specificity, both around 93% according to Herholz et al. (2002). The sensitivity of the PALZ score considerably decreased when applied to our sample of MCI-converter patients and also the choice of a threshold fitted to this sample yielded an overall accuracy around 80%, in line with previous reports in which the PALZ score in MCI-converter patients showed a sensitivity of 79% in a naturalistic population (Galluzzi et al., 2010). The present VOI-based approach associated with an SVM classifier enabled a significantly better performance reaching an accuracy above 90% which is of particular interest when applied to MCI patients. On the other hand, the sensitivity of the PALZ score is based on AD patients reaching 83% in very mild (Herholz et al., 2002) and 85% in mild AD patients (Herholz et al., 2011), but it has not been specifically trained and validated in prodromal AD patients.

Besides the high accuracy achieved by our classification method, its potential interest in everyday clinical routine lies in the implementation of a readily available anatomical atlas and in the use of an in-house Matlab-based script able to assess and sort fully automatically in a few minutes the uptake values in more than hundred segmented VOIs and meta-VOIs making them available for further statistical analyses. The processing time and the use of the script are easier than similar VOI and meta-VOI analyses in which computation time is longer and automation procedures are more complex (Caroli et al., 2012).

The Parahippocampus Gyrus/Amygdala/Hippocampus/Insula complex was found to be among the most valuable 6 meta-VOIs in discriminating MCI-converters from controls both for its uptake values and for asymmetry index. This replicates the findings of Gray et al. who found by SVM, in AD and healthy control cohorts, the right hippocampus and amygdala to be among the few regions showing changes in cross-sectional and longitudinal data, the amygdala being the only region accounting for the difference between stable and converting MCI (Gray et al., 2012). The importance of these structures in the onset and development of AD is well known but it is seldom reported in PET studies using voxel-based analysis tools (such as SPM) possibly due to the contribution of smoothing to the image processing, likely decreasing the possibility to correctly identify and/or segment these small structures (Mosconi, 2005).

On the other hand, although MCI/AD diagnostic criteria usually point to temporo-parietal and posterior cingulate cortex hypometabolisms we also found the frontal and orbitofrontal cortices among the predictors accounting for the highest accuracy both for uptake and asymmetry values. These regions involved in episodic memory encoding and retrieval have been previously described to be hypometabolic in MCI converters in several studies (Drzezga et al., 2003; Nobili et al., 2008) and correlated across studies to memory and cognitive decline being preferentially affected at the time of dementia onset (Anchisi et al., 2005; Caselli et al., 2008; Chen et al., 2010; Herholz et al., 2002; Langbaum et al., 2009; Mosconi et al., 2004b). However, the power of the present analysis including large cohorts of subjects and a sophisticated regional and asymmetry analysis might have allowed the identification of subtle but significant changes already at the early stages of the disease. Frontal and orbitofrontal cortex regions were also grouped by exploratory data analysis into separated components (Nobili et al., 2008; Pagani et al., 2009) and, besides showing significantly lower FDG uptake in MCI converters, in conjunction with a verbal memory test, were among the better discriminators between MCI-converters and healthy controls, with a sensitivity of 82% and a specificity of 100% (Nobili et al., 2008).

The presence of the Postcentral Gyrus/Precentral Gyrus/Supplementary Motor Area complex among the most discriminating meta-VOIs underscores an interesting methodological issue. These regions showed relatively increased uptake at the group level as compared to controls (results not shown), likely due to the semi-quantitative nature of the analyses, that is, in a context in which large portions of the brain have a relatively decreased uptake, preserved regions show a proportionally higher uptake. The higher the difference between affected and spared regions, the higher the probability of neurodegeneration. This is consistent with the study by Chen and coworkers analyzing changes in FDG uptake in MCI and AD patients (Chen et al., 2010). Looking for the best normalization factor to analyze metabolic decline in MCI they found a cluster of spared voxels mainly located in the white matter and somatosensory cortex. In the same study the authors suggested a method to characterize metabolic decline by mean values drawn from the best clusters of voxels, as evaluated in a training set, rather than analyzing predefined anatomical or functional volumes, which might vary in their effect, size and location. We acknowledge that the identification of data-driven regions might improve the appropriateness of data to be analyzed although it implies longer processing time. On the other hand, our method, by taking into account relatively large anatomo-functional VOIs might have reduced the variance of data. Furthermore by decreasing the number of variables and applying a non-linear classification algorithm able to automatically assign an appropriate weight and role to each affected or spared region we obtained a high classification accuracy supporting the validity of the approach.

The evaluation of asymmetries added value to the analyses and fits with the clinical experience in image reading that often highlights differential hemispheric uptake at the very early stages of the disease while bilateral and more symmetric involvement is rather a feature of overt stages (Herholz, 1995). On the other hand a feature of neurodegeneration is a high gradient between more and less affected regions and in this group of MCI-converters the asymmetry index captured metabolic patterns able to yield maximal accuracy. The evaluation of asymmetries along with the ability to capture contrasts between affected and spared regions is the key feature enabling the present SVM model to be sensitive to subtle variation of FDG uptake patterns in the early phase of the disease also in comparison with the PALZ scores, which simply sums the voxel-based t-values in the expected hypometabolic regions.

A rather unexpected result of this study is the lack of a statistically significant effect of age on brain metabolism. The effect of aging on brain blood flow and metabolism is indeed a well known phenomenon, especially in the frontal regions (De Santi et al., 1995). However, such an effect is mainly detectable when all the age ranges are considered, the youngest decades (i.e. from 20 to 50 years of age) clearly showing the highest values but the effect is smoother when considering only middle-aged and elderly subjects. Indeed, in the present study only subjects (controls and patients) in the last decades were included. This may have accounted for the lack of a statistically significant effect.

A limitation of the study is that MCI-converters were discriminated only with respect to healthy controls. Further studies are needed to test if the capability of the used automatic classifier to identify MCI patients about 2 years before conversion will also apply to cohorts of MCI-non-converting to AD or to patients bearer of other confounding pathological conditions. In this respect to properly select the latter groups for statistical analyses a follow-up time as long as 7 years is required (Bennett et al., 2002) and these analyses will be the next step to validate the present methodology. A further limitation of this study relies on the fact that we did not evaluate the possible confounding effect of apolipoprotein E genotype, as these data were available for just some of the patients. Indeed, it has been demonstrated that APOE4 carriers may show peculiar, and more extensive, regions of hypometabolism with respect to the general AD population thus possibly having partially influenced the results of the present analysis (Mosconi et al., 2004a). Similarly, we did not exclude healthy controls whose relatives were affected by dementia and the mean follow-up time of healthy controls was relatively short (i.e. 1 year). Thus, we cannot exclude that someone among controls may have developed cognitive impairment in the following years, as it was shown in previous longitudinal studies (Mosconi et al., 2007). Nevertheless, this would have rather brought on a lower accuracy in distinguishing controls from MCI patients. Finally, the use of different scanners is a further potential limitation of this study, although we tried to minimize any such limitation by using a multiple-factor approach including the balanced number of patients and controls among centers.

In conclusion, in this study we reached a particularly high accuracy in discriminating MCI-converters from healthy controls thanks to the application of a non-linear classifier based on SVM preceded by individuation of anatomo-functional meta-VOIs, normalization to cerebellum and evaluation of inter-hemispheric asymmetries. It has to be underscored that this quite complex and time-consuming data pre-processing was automated and simplified by the in-house created Matlab-based script encouraging its implementation in clinical routine to assist in the diagnosis of AD in aMCI possibly along with MRI and CSF biomarkers.

Even in the era of amyloid PET imaging that has been licensed by regulatory Agencies to exclude rather than to confirm AD, FDG-PET maintains a role not only as a marker of disease evolution but also as an important diagnostic biomarker in the early stages of the disease.

## Conflict of interest

None of the authors declares any conflict of interest.

## Figures and Tables

**Table 1:** Performance measures of the Support Vector Machine classifier as applied to different datasets (value and 95% confidence intervals) and compared with PALZ discrimination analysis tool.

	90R	24R	90R + 45A	24R + 12A	6 R + 6A	PALZ (Th = 11,089)	PALZ (Th = 8116)
Accuracy	.90 (.86–.95)	.85 (.79–.90)	.83 (.77–.89)	.87 (.81–.92)	.91 (.87–.95)	.82 (.75–.87)	.80 (.73–.85)
Sensitivity	.90 (.83–.98)	.84 (.75–.93)	.74 (.63–.85)	.87 (.79–.95)	.92 (.85–.98)	.65 (.52–.75)	.77 (.65–.86)
Specificity	.90 (.84–.96)	.85 (.79–.92)	.88 (.82–.94)	.86 (.80–.93)	.91 (.85–.96)	.92 (.85–.96)	.82 (.73–.88)
Likelihood ratio +	8.95 (5.08–15.78)	5.71 (3.59–9.10)	6.22 (3.66–10.58)	6.33 (3.92–10.22)	10.02 (5.52–18.17)	7.82 (4.07–15.00)	4.21 (2.78–6.41)
Likelihood ratio −	.11 (.05–.23)	.19 (.11–.34)	.29 (.19–.45)	.15 (.08–.29)	.09 (.04–.21)	.39 (.28–.54)	.28 (.17–.44)
Odds ratio	83.15 (29.17–237.0)	30.22 (12.79–71.42)	21.23 (9.43–47.81)	42.30 (16.84–106.2)	112.9 (36.75–346.6)	20.20 (8.57–47.64)	15.26 (7.08–32.88)
Area under curve	.93 (.87–.97)	0.89 (0.82–0.94)	.88 (.82–.93)	.92 (.86–.96)	.95 (.89–.98)	.87 (.80–.91)	.87 (.80–.91)

The classifier was applied to different datasets obtained by different pre-processing of the same set of images.

90R: 90 AAL regions; 24R: 24 anatomo-functional meta-VOIs; 90R + 45A: same as 90R plus inter-hemispheric asymmetries; 24R + 12A: same as 24R plus inter-hemispheric asymmetries; 6R + 6A: 6 meta-regions and 6 inter-hemispheric asymmetries drawn from step-wise backward selection. All parameters, but the area under ROC curve, are dependent on the threshold which was chosen looking for the best point along the ROC curve: considering the PALZ tool the value of the parameter T-sum associated to this point was 8116 (last column) while the standard threshold (implemented in the commercially available software package by PMOD Technologies, Switzerland) is set at 11,090 (second to the last column).
