# Supplementary material for: Volume of interest-based [18F]fluorodeoxyglucose PET discriminates MCI converting to Alzheimer's disease from healthy controls. A European Alzheimer's Disease Consortium (EADC) study
Source: Neuroimage Clin. 2014 Nov 18;7:34–42. doi: 10.1016/j.nicl.2014.11.007 (PMC4299956; doi:10.1016/j.nicl.2014.11.007)
Supplement: Supplementary file 1 — Supplementary material. [file mmc1.pdf]

Supplementary Table e-1. Results of ^{18}F-FDG Brain PET comparison between Healthy Controls and MCI-converting to AD patients

CTR - MCI	p Cluster level	p FWE_{corr}	Maximum Z-score	Region	Talairach coordinates			Cortical region	BA
	0.025	0.001	4.47	Left Cerebrum	-4	-68	46	Precuneus	7
		0.001	4.44	Left Cerebrum	-4	-45	37	PosteriorCingulate Gyrus	31
	0.028	0.001	4.41	Left Thalamus	-4	-17	16	Thalamus	
		0.001	4.38	Right Caudate	10	12	12	Caudate Body	
			4.16	Left Caudate	-18	12	16	Caudate Body	
			4.15	Right Caudate	4	12	3	Caudate Head	
			4.03	Left Caudate	-6	12	5	Caudate Head	
	0.027	0.002	4.25	Left Cerebrum	-50	-48	56	Inferior Parietal Lobule	40
	0.030	0.003	4.13	Left Cerebrum	-34	33	48	Middle Frontal Gyrus	8
		0.005	4.08	Left Cerebrum	-30	44	46	Superior Frontal Gyrus	8
	0.036	0.005	4.08	Right Cerebrum	38	-50	10	Superior Temporal Gyrus	22
		0.005	3.99	Right Cerebrum	46	-30	13	Superior Temporal Gyrus	41
	0.05	0.008	3.90	Right Cerebrum	38.0	-32.0	40.0	Inferior Parietal Lobule	40

CTR: healthy controls (n = 109); MCI: mild cognitive impairment converting to AD (n = 62); BA: Brodmann Area; p FWE_{corr}: $p<0.05$ corrected for multiple comparisons with the Family-wise Error option of SPM
